# Pharmacokinetic and technical comparison of Sandostatin^® ^LAR^® ^and other formulations of long-acting octreotide

**DOI:** 10.1186/1756-0500-4-344

**Published:** 2011-09-09

**Authors:** Holger Petersen, Jean-Claude Bizec, Helmut Schuetz, Marie-Laure Delporte

**Affiliations:** 1 Novartis Pharma AG, Basel, Switzerland

## Abstract

**Background:**

Sandostatin^® ^LAR^® ^(Novartis Pharma AG) is a long-acting repeatable formulation of the somatostatin analogue octreotide, the safety and efficacy of which has been established through 15 years of clinical experience. Recently, other formulations of octreotide using polymer poly(lactic-co-glycolic acid) technology have been developed. This study compares the composition and pharmacokinetic (PK) profile of Sandostatin LAR with three other versions of the depot delivery system (formulations A, B and C, available in selected countries).

**Findings:**

Sandostatin LAR exhibited a characteristic concentration-time profile with a limited initial release of octreotide ('burst'), an erosion phase from weeks 3-5, and a slowly declining concentration to day 52. The PK profiles of formulations A and B were characterized by a large initial burst during days 0-2, with up to 41% of the overall area under the plasma-concentration time curve achieved. Low and variable octreotide concentrations were observed during the microparticle erosion phase (days 2-62 [day 82 formulation C]) for formulations A, B and C. Sandostatin LAR microparticles are spherical in shape with an average diameter of approximately 50 μm, determined by scanning electron microscopy evaluation. Formulation A had smaller, irregular microparticles, and formulations B and C exhibited a large range of particle diameters (< 20 to > 100 μm). Inductively coupled plasma-optical emission spectroscopy detected a high tin content of 104 mg/kg in formulation B, the presence of which may suggest inadequate purification following polymer synthesis using tin(II)-octoate as catalyst. PK profiles for formulations A, B and C after a single intramuscular injection of 4 mg/kg in male New Zealand rabbits differed markedly from the PK profile of Sandostatin LAR.

**Conclusions:**

Clear differences were seen between Sandostatin LAR and formulations A, B and C, including variations in microparticle size, shape and impurity content. Considering the significant differences in the octreotide release profile between Sandostatin LAR and the other formulations, the safety and efficacy of the other formulations cannot be inferred from the Sandostatin LAR efficacy and safety profile; each of these other formulations should be assessed accordingly.

## Findings

### Background and aims

Sandostatin^® ^LAR^® ^is a long-acting octreotide formulation for the treatment of patients with acromegaly and symptoms associated with certain types of neuroendocrine tumors. Approved at doses of 10, 20 and 30 mg (and up to 40 mg for patients with acromegaly in certain countries such as the US and Japan), Sandostatin LAR allows for once-monthly administration, maintaining the efficacy of Sandostatin immediate-release whilst significantly reducing the number of injections administered [1,2]. Based on a well defined and consistent pharmacokinetic (PK) profile, the efficacy and safety of Sandostatin LAR have been established over more than a decade of clinical experience [3].

Recently, other long-acting octreotide formulations have become available for clinical use in selected markets. Evidence regarding bioequivalence or product property equivalence between these new formulations and Sandostatin LAR is not available. Such information is important as clinical guidelines recommending the use of sustained-release octreotide are based on experience with Sandostatin LAR, and assumes that other long-acting octreotide formulations would be of a similar quality, uniformity and reliability. Here we report findings from a series of assessments performed to compare Sandostatin LAR with three other formulations of long-acting octreotide manufactured by companies other than Novartis.

The properties of Sandostatin^® ^LAR^®^, and those of three other long-acting octreotide products, were quantitatively and qualitatively assessed. Formulations A, B and C were manufactured from 2008-2009. The evaluations aimed to compare the composition and physico-chemical properties of the other formulations with those of Sandostatin LAR. PK data were compared using a rabbit model. The different formulations were tested in three separate animal studies of similar study design that allowed for comparison of data.

## Methods

### Microparticle appearance and composition

Samples were analyzed according to cGLP in an unblinded manner. Scanning electron microscopy (Zeiss Supra 40) was used to evaluate microparticle size, shape, porosity and surface appearance. Samples were sputtered with gold-palladium prior to analysis. For evaluation of cross-sections, microparticles were embedded in an epoxy resin, polymerized and fractured in liquid nitrogen before sputtering with gold-palladium. Proton nuclear magnetic resonance (^1^H-NMR) was used to assess the composition of each formulation and to determine the ratio of lactide to glycolide within the poly(lactic-co-glycolic acid; PLGA) polymer. Samples of 1.1-1.3 mg dissolved in 0.5 mL dimethyl-d_6 _sulfoxide (DMSO-d_6_) were analyzed with 500-600 MHz ^1^H-NMR for 12 hours at ambient temperature. Gel-permeation chromatography with IR detection was used to determine the molecular weight of the PLGA polymer against polystyrene standards. Inductively coupled plasma-optical emission spectroscopy (ICP-OES; limit of detection 1 mg/kg) was used to detect tin levels in the polymer by measuring any residual presence of the tin(II)-octoate catalyst. The sample was decomposed at 250°C in a closed pressurized system and signals were quantified versus an external calibration function.

### Analysis of PK parameters

Sandostatin LAR, as well as formulations A, B and C, were administered as single intramuscular injections at a nominal dose of 4 mg/kg to male New Zealand white rabbits aged 3.5-4 months and weighing 3.00-3.46 kg (four cohorts, n = 3, 4 or 7 rabbits per group). For formulations A and B, the absolute octreotide content in the vials supplied was stated by the manufacturer to be 20 mg. As such, concentrations were calculated assuming 20 mg of octreotide per vial; verification of the octreotide content was not performed. For formulation C, rabbits were given a target dose of 10 mg/rabbit. Actual doses are reported in Table 1. Rabbits were housed in single cages with elevated sitting boards, and allowed free access to standard rabbit and guinea pig chow and water. Environmental conditions were 19 ± 2°C with 55 ± 15% humidity.

**Table 1:** PK parameters of Sandostatin^® ^LAR^**®**^, and formulations A, B and C

PK parameters	Sandostatin® LAR^®^	Formulation A	Formulation B	Formulation C
Number of rabbits	7	4	3	3
Actual dose, mg/kg (± SD)	3.34 ± 0.236	3.42 ± 0.410	3.00 ± 1.84	1.27 ± 0.426
Burst phase, days 0-2				
t_max-burst_, hours, median	2	1.0	0.5	0.02
C_max-burst_, ng/mL, mean (± SD)	3.49 ± 4.21	167 ± 31.1^**†**^	22.8 ± 18.0	20.5 ± 6.64
Erosion phase, days 2-62*				
t_max-erosion_, days, median	20	14 and 34	20.0	12
C_max-erosion_, ng/mL, mean (± SD)	9.97 ± 4.21	3.89 ± 1.73, and 2.42 ± 2.11	4.83 ± 3.91	13.3 ± 3.52
AUC_0-last_, d·ng/mL (± SD)*	179 ± 62.0	163 ± 33.7	86.1 ± 73.2	247 ± 33.4
AUC_0-2d_, d·ng/mL (± SD)	4.42 ± 5.70	65.3 ± 4.57	2.61 ± 0.680	2.39 ± 0.592
Burst, % (± SD)	2.19 ± 1.97	41.0 ± 6.32	22.2 ± 35.0	1

*The AUC_0-last _was measured over 52 days (AUC_0-52d_) for Sandostatin LAR, over 62 days (AUC_0-62d_) for formulations A and B, and over 82 days (AUC_0-82d_) for formulation C.

^†^C_max-burst _of formulation A is significantly different to that of Sandostatin LAR.

AUC, area under the plasma concentration-time curve; C_max_, maximum plasma concentration; SD, standard deviation; t_max_, time to C_max_.

Blood collection was taken pre-dose, and then up to 52, 62 or 82 days post-dose. Plasma concentrations of octreotide were determined by radioimmunoassay with a lower limit of quantification of 0.05 ng/mL. Area under the plasma concentration-time curve (AUC), maximum plasma concentration (C_max_) and time to C_max _(t_max_) were evaluated. C_max _and t_max _values were calculated in both the burst phase from days 0-2 (C_max-burst_; t_max-burst_) and erosion phase from days 2-last sampling time (C_max-erosion_; t_max-erosion_).

Animal research was performed in accordance with international guidelines and follows the Swiss law for animal experimentation.

### Statistical methods

All PK parameters were calculated with WinNonlin software version 5. PK parameters were calculated using a non-compartmental model. AUC from days 0 to the last sampling time (AUC_last_, i.e. AUC_0-52d_, AUC_0-62d _and AUC_0-82d_) was calculated using the linear trapezoidal rule. Percentage of burst from days 0-2 (AUC_0-2d_) was calculated as: 100 × AUC_0-2d_/AUC_last_. One-way analysis of AUC and C_max _was performed using the Kruskal-Wallis method when data were non-parametric.

## Results

### Microparticle size, shape, porosity and surface appearance

**Figure 1:**
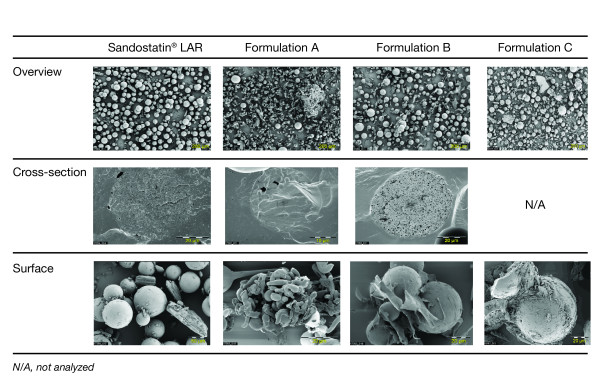
**Scanning electron microscopy comparisons of Sandostatin^® ^LAR^®^, and formulations A, B and C**.

Sandostatin^® ^LAR^® ^microparticles were spherical in shape with approximately 50 μm diameter on average (Figure 1; Overview). In contrast, formulation A microparticles had a much smaller diameter and were of irregular shape. The microparticles in formulations B and C were mostly spherical in shape and had a similar mean diameter to the Sandostatin LAR microparticles, however, a larger range of particle diameters was observed (< 20 to > 100 μm). Furthermore, some microparticles in formulation C had an irregular shape and appeared to be damaged (Figure 1; Overview). Sample cross-sections of the microparticles revealed that the Sandostatin LAR microparticles were very compact with only minor pores, microparticles in formulation A had no pores and those in formulation B had a very high porosity (approximately 1-3 μm diameters: Figure 1; Cross-section). There was an insufficient quantity of formulation C available for a cross-sectional analysis.

**Figure 2:**
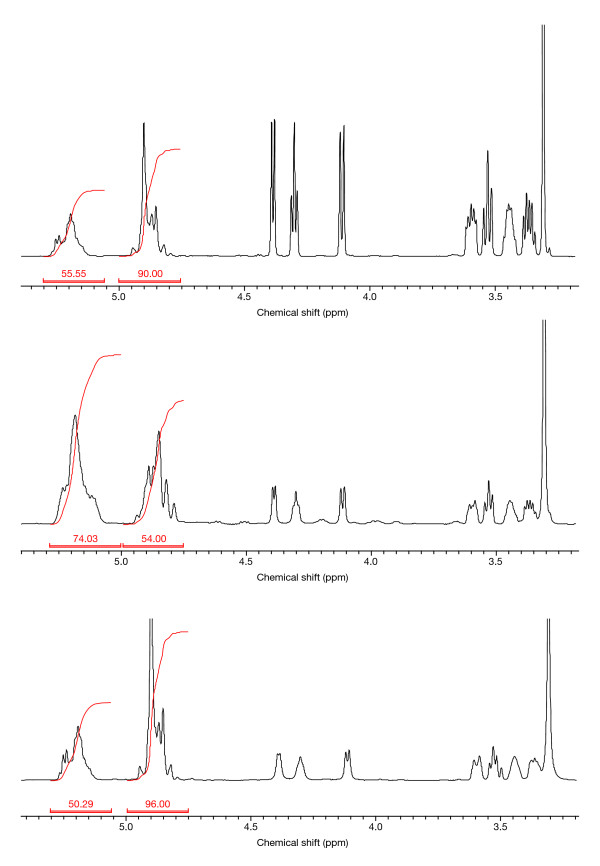
**^1^H-NMR of Sandostatin^® ^LAR^®^, formulation A and formulation B**.Upper spectrum: Sandostatin LAR, molar ratio actide 55, glycolide 45. Middle spectrum: formulation A, molar ratio lactide 73, glycolide 27. Lower spectrum: formulation B, molar ratio lactide 50, glycolide 50. Signals at 5.2 ppm indicate the single methine proton of the lactide monomer. Signals at 4.8 ppm are assigned to the two methylene protons of the glycolide monomer. Signals at lower shifts than 4.5 ppm indicate mannitol (mannitol-OH and -CH/CH2).

Sandostatin LAR and formulations A, B and C incorporate mannitol as a bulk excipient. NMR evaluation confirmed the presence of mannitol (Figure 2). Inspection of the microparticle surface of lyophilized samples revealed differences in the mannitol appearance among the formulations. Mannitol was found in a crystalline shape loosely connected to the microparticles in the Sandostatin LAR formulation. In contrast, mannitol typically had a non-crystalline shape in formulations A and B. The mannitol in formulation A was also more porous and of a smaller size than that in Sandostatin LAR. In formulation C, mannitol particles were very large and irregularly shaped (Figure 1; Surface).

### Molecular composition

The prescribing information for Sandostatin LAR, and formulations A and C, lists acetate as the octreotide salt; the salt type was not stated in the prescribing information for formulation B. ^1^H-NMR analysis of formulations A and B suggested the presence of an acetate component. Compared with Sandostatin LAR, formulations A and B exhibited a lower molar ratio between the acid and base of the octreotide salt (16% and 75% of that ratio determined for Sandostatin LAR [100%], respectively). ^1^H-NMR analysis was not performed on formulation C due to an insufficient quantity of sample.

### Molecular weight of the polymer

Differences were observed between the molecular weight and composition of the polymer used in Sandostatin LAR, and formulations A, B and C. Whereas the molecular weight of the Sandostatin LAR polymer was 52 kDa, formulations A, B and C had lower molecular weights of approximately 16, 32 and 14.5 kDa, respectively. The ratio of the lactide:glycolide co-monomers was 55:45 in Sandostatin LAR, 73:27 for formulation A and 50:50 for formulation B. For formulation C, the supplier claimed a ratio of 62.5:37.5 based on a 1:1 blend of 50:50 and 75:25 PLGA polymers.

### Impurities

No heavy metals or other potentially toxic substances were detected in Sandostatin LAR, or in formulation A. A high tin content of 104 mg/kg was found in formulation B. ICP-OES analysis was not performed on formulation C due to an insufficient quantity of sample.

### Comparison of PK profile in rabbits

**Figure 3:**
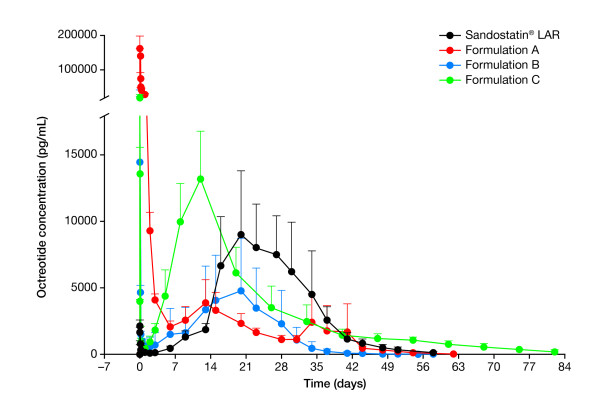
**Plasma concentration-time profiles ± SD of Sandostatin^® ^LAR^®^, and formulations A, B and C**.

Sandostatin LAR demonstrated controlled release of octreotide. During the burst phase (days 0-2) the mean C_max-burst _value was 3.49 ng/mL, corresponding to 1.9% of the overall AUC_0-52d_. The octreotide concentration increased during the erosion phase to reach mean peak levels of 9.97 ng/mL (Figure 3; Table 1). The release pattern of Sandostatin LAR demonstrated an erosion phase in octreotide concentrations during weeks 3-5, similar to the concentration-time profile observed in humans [4].

Compared with the PK profile of Sandostatin LAR, formulations A, B and C achieved a much higher octreotide concentration over the first 2 days post injection, displaying mean C_max-burst _of 167, 22.8 and 20.5 ng/mL, respectively. Although C_max-burst _was high for formulation C, AUC_0-2d _was relatively low. During the first 2 days after injection, 41.0% and 22.2% of the overall AUC_0-62d _was observed with formulations A and B, respectively. Consequently, during the erosion phase, octreotide concentrations in these formulations were much lower and more variable compared with those of Sandostatin LAR; these formulations had no obvious plateau phase (Figure 3). Overall octreotide concentrations were also found to be lower with formulations A and B. Whereas the Sandostatin LAR samples demonstrated an AUC_0-52d _of 179 d·ng/mL, formulation A and B demonstrated AUC_0-62d _of 163 and 86.1 d·ng/mL, respectively, and formulation C demonstrated AUC_0-82d _of 247 d·ng/mL. Additionally, formulation B was found to be very difficult to inject due to needle clogging and, as a result, one rabbit was excluded as no octreotide concentration was detected after an unsuccessful injection. A second rabbit had an octreotide concentration considerably lower than the mean dose exhibited in the other cohorts (23.2% of the intended dose, individual data not shown). Concerning formulation C, early erosion was observed with t_max-erosion _of 12 days with a high C_max-burst _of 20.5 ng/mL.

## Discussion

Sandostatin^® ^LAR^® ^is a long-acting repeatable formulation of octreotide. Its development required extensive analytical support to ensure the quality and consistency of the formulation. Clinical PK studies have established that Sandostatin LAR produces a reliable, sustained release of octreotide [4,5], with proven therapeutic utility in patients [3,6,7]. More recently, other long-acting formulations of octreotide have been introduced in selected markets.

In humans, Sandostatin LAR has a well-characterized consistent and predictable PK profile, which can be described as exhibiting three distinct phases: (1) release of surface-absorbed octreotide (burst); (2) pore diffusion, biodegradation, osmotic swelling and ionic interactions (erosion phase leading to a drug concentration plateau); and (3) fragmentation and complete biodegradation of the polymer (erosion phase leading to complete drug release) [8]. This tripartite pattern has been regularly observed and is evident with various Sandostatin LAR doses. Octreotide concentrations exhibited an initial peak on day 1, followed by a decline over the following 3-5 days, before slowly increasing and reaching a plateau 2-3 weeks post injection before declining [4]. The steady-state PK simulation of Sandostatin LAR 20 mg suggested a mean concentration of 1216 pg/mL (range, 1065-1585 pg/mL) with a fluctuation index of 43%. Additionally, inter-subject variability in mean C_max _was 32% for Sandostatin LAR 20 mg [4].

During the *in vivo *rabbit PK evaluations in the present study, differences in the concentration-time profile between formulations A, B and C, and Sandostatin LAR, were observed. The Sandostatin LAR concentration-time profiles in these *in vivo *investigations were similar to those observed in humans [4]. During the burst phase, the three other formulations displayed AUC_0-2d _values ranging from 2.39-65.3 d·ng/mL, compared with 4.42 d·ng/mL for Sandostatin LAR. This variability may result from the appearance of the microparticles, and poses potential safety risks. This finding was particularly evident in formulation A, with 41% of the overall AUC achieved within the first 2 days after injection. Formulations A and B also demonstrated much lower concentrations of octreotide, while formulation C was characterized by an early and narrow erosion phase with no discernable plateau. As such, in addition to potential safety concerns related to the large burst phase, the formulations may also fail to consistently deliver therapeutic concentrations of octreotide to patients throughout the interval between injections.

It is important to note the constraints of our study that limit the interpretation of our findings. First, *in vivo *rabbit PK data do not always accurately reflect, and cannot replace, clinical PK studies in humans. A rabbit PK profile similar to that of Sandostatin LAR is not proof of clinical bioequivalence to Sandostatin LAR and cannot replace demonstrating human bioequivalence. This underlines the importance of performing clinical PK studies in all new depot delivery systems of octreotide. Second, clinical studies have to demonstrate equivalent safety and efficacy in specific indications; target patient populations include those with acromegaly or neuroendocrine tumors. In addition, although the *in vivo *study described here was designed to evaluate the formulations in an equal number of rabbits per cohort, needle clogging in formulation B caused one animal to be excluded from analysis and one animal to receive part of the intended sample amount. Furthermore, only a small quantity of formulation C was available and, therefore, this sample could be evaluated only in three rabbits. A further study with a larger sample size would strengthen the evidence presented here. Finally, disparities in study design should be taken into account: differences in serum sample time points between formulations occurred because of resource availability and the fact that the *in vivo *evaluations of different formulations were performed on different calendar dates. Nevertheless, it is reasonable to compare the kinetic profile of the formulations because the serum sample times covered the long *in vivo *release profile expected in these products.

Sandostatin LAR consists of octreotide acetate encapsulated and uniformly distributed within PLGA D-(+) glucose microspheres. Slow release of the drug occurs as the polymer biodegrades, primarily through hydrolysis. The polymer has an average molecular weight of ~52 kDa and the microparticles exhibit a mean diameter of ~50 μm [8].

Compared with the established characteristics of Sandostatin LAR, formulations A and C exhibited greater irregularity in microparticle shape and size. This is suggestive of inadequately encapsulated octreotide molecules and may indicate a lack of quality control in the manufacturing process. Variations in the diameter of the microspheres and the thickness of the polymer coat in formulations B and C have the potential to affect the drug-release profile [9], with possible failure to deliver continuous therapeutic drug concentrations, and/or can potentially cause adverse events related to excessive drug release during the initial burst phase. In addition, differences in the mannitol appearance were observed between the formulations. Since mannitol is used to improve flow and dispersability and to improve stability in drug delivery systems, it could be postulated that changes in its appearance could affect the pharmaceutical processability of PLGA-based drug delivery systems as well as the preparation of the drug for administration.

Factors such as the molecular weight and composition of the PLGA polymer also affect drug release, with low molecular weight accelerating the rate of drug release and a high lactide:glycolide ratio causing the polymer to degrade more slowly because the lactide monomer is more hydrophobic than the glycolide monomer. In previous studies of octreotide release from PLGA polymers of various molecular weights and lactide:glycolide ratios, pH and impurity content also influenced the percentage of octreotide release [10,11]. Although the very low molecular weight in formulations A and C may be in part offset by a higher amount of lactide monomer, the differences in molecular weights and lactide:glycolide ratios between the three formulations are likely to cause different octreotide release patterns. As PLGA polymers are routinely used in sustained-release formulations and can be manufactured to a much higher purity than that present in formulation B, the polymer can be considered to be of poor quality. Variability was further evident in the porosity of microparticles in formulations A and B. Previous studies have found that biodegradation and drug release are dependent on the porosity, with variations affecting the rate of drug mobility [12].

The high tin concentration found in formulation B may indicate that high amounts of tin(II)-octoate were used in the polymer synthesis without proper purification, likely to be due to residual product from the catalyst used during production of the polymer. As tin(II)-octoate has been reported to be highly cytotoxic,[13] this may affect patient safety. This impurity was not observed in Sandostatin LAR or formulation A and no arsenic content was found in any sample. Quality control to guarantee these characteristics is paramount to LAR formulations. In addition, formulations A and B had a low acid component to the octreotide salt. Theoretically, in the case of an acid-base pair, the ratio of acetate molecules to octreotide molecules should be 2:1. In these formulations, octreotide is likely to be present as a free base rather than as an acetate salt indicating that the other formulations do not share the same product characteristics as Sandostatin LAR.

In conclusion, clear differences were seen between Sandostatin LAR and formulations A, B and C, including significant differences in the PK profile, and variations in microparticle size, shape molecular weight, acid:base ratio, and impurity content. These findings suggest that other long-acting octreotide formulations may have a different drug-release pattern to that of Sandostatin LAR despite similar composition. Considering these differences, formulations A, B and C are most likely not bioequivalent to Sandostatin LAR in humans. Consequently, the safety and efficacy of these new formulations cannot be inferred from the Sandostatin LAR clinical and safety profile. Each of these other formulations should be assessed by appropriate clinical studies to determine their clinical benefit and safety profiles.

## List of abbreviations

AUC: area under the plasma concentration-time curve; AUC_last_: AUC from days 0 to the last sampling time; C_max_: maximum plasma concentration; C_max-burst_: maximum octreotide concentration during the burst phase from days 0-2; C_max-erosion_: maximum octreotide concentration during the erosion phase from days 2-62; DMSO-d_6_: dimethyl-d_6 _sulfoxide; ^1^H-NMR: proton nuclear magnetic resonance; ICP-OES: inductively-coupled plasma-optical emission spectroscopy; kDa: kilodalton; LAR: long-acting repeatable; PLGA: poly(lactic-co-glycolic acid)**; **PK: pharmacokinetic; t_max_: time to C_max; _t_max-burst_: time to C_max-burst; _t_max-erosion_: time to C_max-erosion_

## Competing interests

This study was funded by Novartis Pharma AG. HP, J-CB, HS and M-LD are employees of Novartis Pharma AG.

## Authors' contributions

J-CB, HS and M-LD participated in performance and interpretation of the PK analysis, and HS and M-LD wrote the PK analysis report on which this manuscript is based. HP designed and performed the technical quality assessment tests and wrote the report on which the non-PK aspects of this manuscript are based. All authors helped to critically revise manuscript drafts. All authors read and approved the final manuscript.
